# Removal efficacy of propolis/calcium hydroxide medicaments from the root canal

**DOI:** 10.12669/pjms.37.7.4241

**Published:** 2021

**Authors:** Muhammad Adeel Ahmed

**Affiliations:** 1 Muhammad Adeel Ahmed Department of Restorative Dentistry and Endodontics, College of Dentistry, King Faisal University, Al-Ahsa, Saudi Arabia

**Keywords:** Calcium hydroxide, Propolis, Removal potency, Teeth root canal

## Abstract

**Objective:**

To compare the removal efficacy of propolis and calcium hydroxide medicaments from the root canal using manual irrigation with sodium hypochlorite.

**Methods:**

A randomized controlled trial was conducted at the Department of Restorative Dentistry and Endodontics, College of Dentistry, King Faisal University for two months. Thirty single-rooted upper or lower permanent anterior teeth with sound root and closed apex were selected. After cleaning and shaping of canal by protaper rotary, teeth were randomly divided into two groups. In group “A,” propolis paste was placed while calcium hydroxide was placed inside root canals in group “B,” followed by temporary restoration. After seven days, intracanal medicament was removed using 25 # K file and irrigated canal by 5 ml of 3.0% sodium hypochlorite. A final irrigation of 2-ml of 17% EDTA for three minutes followed by 1-ml of normal saline was performed. A diamond disc was used to cut the crowns of the teeth from cemento-enamel junction and divide the roots into two halves. These sectioned halves were then observed under a stereomicroscope at 7X magnification and analyzed using Adobe Photoshop. Magnetic lasso tool was used to calculate the percentage of residual medicament in the canals by comparing the pixel proportion of the medicament with the total pixel proportion of the canal. Paired t-test was used to see the difference in the number of remaining remnants between propolis and calcium hydroxide. A p-value of less than 0.5 was considered statistically significant.

**Results:**

A statistically significant difference (p-value < 0.001) was observed in the percentage of remaining remnants between propolis (23.22%) and calcium hydroxide (38.58%) after thorough irrigation with sodium hypochlorite.

**Conclusion:**

Propolis is superior to calcium hydroxide in terms of their removal potency from the root canal after thorough irrigation with sodium hypochlorite.

## INTRODUCTION

The success of endodontic treatment relies on proper cleaning and disinfection of the root canal from infectious microorganism.^1^ Calcium hydroxide is most commonly used intracanal medicament aimed to achieve root canal disinfection; however, it is not very effective in root canal re-treatment cases in which *Enterococcus faecalis (E. faecalis)* is predominant microorganism.^2^ In such scenario, various other intracanal medicaments have been recommended to eradicate *Enterococcus faecalis* such as chlorhexidine, ledermix and triple antibiotics paste.^3^

One of the newly emerging, natural intracanal medicament is propolis. Propolis is wax-cum-resin substance prepared by honeybees to protect their honey from contamination of microorganism.^4^ It has an array of anti-bacterial, anti-oxidant, anti-inflammatory properties, which render its use for multiple purposes in dentistry.^4^ Numerous studies have recommended the use of propolis as an intracanal medicament due to its effectiveness against *E. faecalis*.^5^,^6^ The mechanism of action of propolis is based on its effects on membrane permeability and membrane potential of *E. faecalis*.^6^ Propolis was also reported to remain unchanged by the buffering capacity of dentin in contrast to calcium hydroxide.^7^ Awawdeh et al.^8^ and Victorino et al.^9^ showed that propolis was more efficient than calcium hydroxide against *E. faecalis* while Madhubala et al.^10^ also reported the efficacy of propolis as 100% against *E. faecalis* following a 7-day application.

Retrievability of intracanal medicament is as important as their placement inside canal.^11^ Several studies have shown that complete removal of intracanal medicament is utmost important to achieve good quality obturation without voids.^12^–^13^ In addition, it is clinically evident that the presence of intracanal medicament such as calcium hydroxide can interact chemically with zinc oxide eugenol based sealers, interferes with sealer adhesion to the dentinal wall and halt its penetration in to dentinal tubules thus increases the likely chances of root canal treatment failure due to leakage.^14^ As far as propolis is concerned, knowledge of their retrievability when used as an intracanal medicament is scarce in medical literature. Therefore, this study investigated the removal efficacy of propolis compared to calcium hydroxide paste using manual irrigation with sodium hypochlorite. This study has explored the opportunity of using natural intracanal medicament as an alternative to traditional medicament in relation to an issue of difficult retrievability of medicament from the root canal. Moreover, it has opened an avenue for further research in this direction.

## METHODS

A randomized controlled trial was conducted at the Department of Restorative Dentistry and Endodontics, College of Dentistry, King Faisal University, Al Ahsa for two months. The study approval was obtained from Deanship of Scientific Research, King Faisal University in November 2020 (KFU-REC/2020-11-20). Thirty single-rooted upper or lower anterior permanent teeth with sound root and closed apex were selected. Teeth with signs of root resorption, perforation, severe root curvature and root caries were excluded. The selected teeth were steam autoclaved (Vacuklav 30B+, MELAG, Berlin, Germany) at 121ºC at 15 psi for 30 minutes and were stored in tap water containing 0.1% thymol. Initially, the access cavity was prepared using round bur (Mani, Japan) in high-speed hand piece, then working length was established using 25# K file (Mani, Japan). After then root canal cleaning and shaping was performed using Protaper rotary file (DENTSPLY, Switzerland) along with copious irrigation of 3.0% sodium hypochlorite. Finally, canals were dried with paper points and teeth were randomly and equally divided into two groups by the lottery method.

### Group-A (Study group)

Propolis paste was placed in the root canal with the help of lentulo-spiral in slow speed hand piece up to the working length until the medicament extruded out of the apex and backfilled to the level of the canal orifice followed by cotton and temporary restoration.

### Group-B (Control group)

Calcium hydroxide paste (Metapaste, Meta Biomed, Chungcheongbu

k-do, Korea) was placed in the root canal similarly like Group-A.

**Fig.1:**
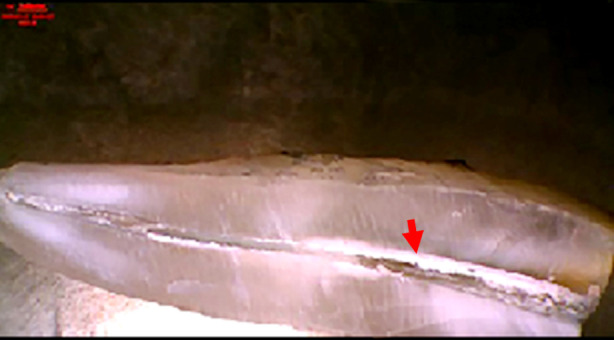
Remaining 38.58% calcium hydroxide in the canal after final irrigation.

**Fig.2:**
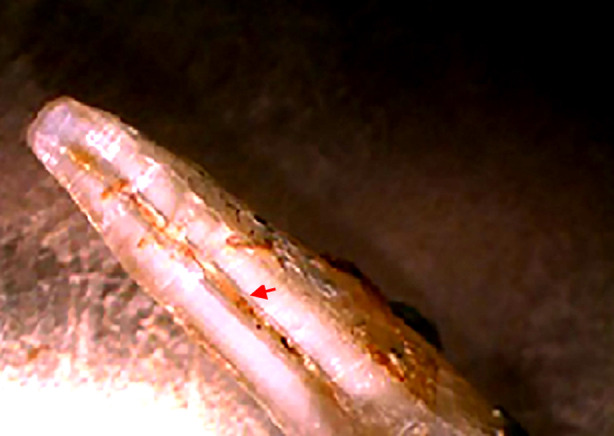
Remaining 23.22% propolis in the canal after final irrigation.

After seven days, access to the root canal regained by removing temporary restoration. Intracanal medicament was removed using 25 # K file and canal irrigation with 5 ml of 3.0% sodium hypochlorite (CanalPro NaOCl, Coltène/Whaledent, Altstätten, Switzerland). A final irrigation of 2-ml of 17% EDTA (CanalPro EDTA, Coltène/Whaledent, Altstätten, Switzerland) (Ethylenediamine tetraacetic acid) for three minutes followed by 1-ml. of normal saline was performed. A diamond disc was used to cut the crown of the teeth from cemento-enamel junction and divide the root into two halves. These sectioned halves were then observed under a stereomicroscope (BOECO, Hamburg, Germany) at 7X magnification and analyzed using Adobe Photoshop (Version 19, Adobe, California, USA) by the principal investigator (Fig.1 and 2). Magnetic lasso tool was used to calculate the percentage of residual medicament in the canal by comparing the pixel proportion of the medicament with the total pixel proportion of the canal.

All data were entered in SPSS (Version 25.0. Armonk, NY, USA). Paired t-test was used to see the difference in the number of remaining remnants between propolis and calcium hydroxide. A *p*-value of less than 0.5 was considered statistically significant.

## RESULTS

The results of the current study show statistically significant difference (p-value *=* 0.001) in the percentage of remaining remnants in the root canal between calcium hydroxide and propolis after thorough irrigation with sodium hypochlorite. The percentage of remaining remnants were calculated by highlighting the total area of the canal and the area covered by intracanal medicament. The resultant values for areas covered by intracanal medicament were divided by their respective total canal areas and the percentages were drawn.

The descriptive statistics and paired t-test values after analysis through SPSS version 25 is shown in Table-I. The working hypothesis was that there is a difference between removal efficacy of propolis and calcium hydroxide intracanal medicament after manual irrigation of root canals by 3.0% sodium hypochlorite followed by final irrigation with EDTA (Ethylenediamine tetraacetic acid) and normal saline. Hence, during in depth analysis calcium hydroxide remnants were found abundant (38.58%) in the root canals compared to propolis 23.22% after irrigation. Both agents showed a significant difference in terms of removal potency when paired t-test was applied to the mean values of both groups. (*p*-value = 0.001).

**Table I:** Amount of remaining intracanal medicament in the root canal.

*Groups*	*Mean residual value n%*	*Sth. Deviation*	*Mean difference n %*	*P-value*
Remaining Propolis	23.22	5.46132	15.36	0.001
Remaining Calcium Hydroxide	38.58	4.81355		

## DISCUSSION

The study was conducted to compare the removal efficacy of propolis and calcium hydroxide paste from root canal using manual irrigation with sodium hypochlorite. The results of the current study show that the amount of remaining calcium hydroxide remnant in root canal after thorough irrigation with sodium hypochlorite was more than propolis.

Rouhani et al.^15^ found similar results in which the residual amount of calcium hydroxide on the canal walls was significantly higher than that of propolis (P=0.001). They used UMAX scanner and microstructure image processing software for the evaluation of residual residue of calcium hydroxide and propolis in comparison stereomicroscope at 7X magnification used in the current study.

Contrarily, Victoria et al.^16^ found no statistically significant difference in the amount of remaining remnant and concluded that difficulty in removing propolis from the canal was similar to that observed for calcium hydroxide. This dissimilarity in result may be due to differences in the method of remaining material evaluation, tooth type (open or closed apex) and the difference in the form of propolis used.

Several studies support the use of calcium hydroxide intracanal medicament owing to its advantages such as it produces anti-bacterial effects, promote healing and repair, neutralize low pH acids and stops internal resorption.^17^,^18^ However, it could be potentially toxic due to its high pH and can cause cellular necrosis or chronic inflammation in the periapical ligament if extruded from the canal to periapical area during clinical use.^19^

Complete removal of intracanal medicament is always desirable. Numerous studies have shown that the presence of remaining intracanal medicament in the canal before obturation may disturb sealer adhesion, create voids in the canal and chemically interact with zinc oxide eugenol-based sealers and produce calcium eugenolate.^20^,^21^

Factors that favor retention of intracanal medicament in the canal for instance; viscosity, water or oil-based vehicle may in some way become unfavorable when it comes to its retrieval.^22^ Recently, new advancement in irrigation equipment and technique such EndoVac, EndoActivator and RinsEndo has improved the efficiency of canal cleaning, but none of the technique observed complete removal of calcium hydroxide paste from the canal.^23^,^24^ However, some machine-assisted methods are more efficient than manual irrigation.^25^

The findings of the current study emphasized that complete removal of calcium hydroxide intracanal medicament is not achievable with conventional filing and canal irrigation by sodium hypochlorite. On the other hand, propolis removal was not absolute but is achieved in a significantly higher amount. Hence, propolis could be used alternatively to calcium hydroxide intracanal medicament. In addition, this study has opened an avenue for further research in this direction.

### Limitations of the study

The findings of the current study must be seen considering some limitations. First, the manual irrigation technique was used for cleaning the root canal from debris and intracanal medicaments instead of machine-assisted techniques. Second, propolis is available in different forms in the market, the removal efficacy of another form might be different; therefore, the results cannot be generalizable. Hence, further studies are recommended to compare and evaluate intracanal medicament removal by advanced irrigation methods and techniques.

## CONCLUSION

Propolis is superior to calcium hydroxide in terms of their removal from the root canal after thorough irrigation with sodium hypochlorite.
